# Retraction: Superior Mesenteric Artery Thrombosis Following Severe COVID-19 Pneumonia

**DOI:** 10.7759/cureus.r103

**Published:** 2024-01-25

**Authors:** Abdulhadi A Alali, Mohammed O Baqais, Fayez M Albishi, Asmaa I Alkhamis, Yusuf A Alshehri, Khadijah F Amri, Rana F Albenayan, Shifa A Khudeer, Muayad M Anbarserri, Mohammed S Alsharif, Safiah M Hakami, Manar A Bahammam, Noor J Altooq, Faisal Al-Hawaj

**Affiliations:** 1 College of Medicine, Vision Colleges, Riyadh, SAU; 2 College of Medicine, King Saud University, Riyadh, SAU; 3 College of Medicine, King Khalid University, Abha, SAU; 4 College of Medicine, King Faisal University, Hofuf, SAU; 5 College of Medicine, King Abdulaziz University, Jeddah, SAU; 6 College of Medicine, Jazan University, Jazan, SAU; 7 Emergency Department, Primary Health Care Center Al-Awali, Al-Awali, SAU; 8 Emergency Department, Primary Health Care Center Al-Aziziyah, Al-Aziziyah, SAU; 9 College of Medicine, Imam Mohammad Ibn Saud Islamic University, Riyadh, SAU; 10 College of Medicine, AlMaarefa University, Ad-Diriyah, SAU; 11 Medicine, Prince Mohammed Bin Nasser Hospital, Jazan, SAU; 12 College of Medicine, Imam Abdulrahman Bin Faisal University, Dammam, SAU; 13 College of Medicine, Arabian Gulf University, Manama, BHR

The Editors-in-Chief have retracted this article. Concerns were raised regarding the identity of the authors on this article. Specifically, Faisal Alhaway and Malak Shammari have stated that they were added to this article without their knowledge or approval. The identity of the other authors could also not be verified. As the appropriate authorship of this work cannot be established, the Editors-in-Chief no longer have confidence in the results and conclusions of this article.

